# SID Trp–Lys Ratio on Pig Performance and Immune Response After LPS Challenge

**DOI:** 10.3390/ani15091194

**Published:** 2025-04-23

**Authors:** Stephane Alverina Briguente Da Motta, Nathana Rudio Furlani, Antonio Carlos Lourenço, Sergio Xavier Silva Junior, Juliana Cristina Ramos Rezende, Melissa Izabel Hannas

**Affiliations:** 1 Department of Animal Science, Federal University of Viçosa, Viçosa 36570-900, Minas Gerais, Brazil; stephane.motta@ufv.br (S.A.B.D.M.); nathana.furlani@ufv.br (N.R.F.); antonio.lourenco@ufv.br (A.C.L.); sergio.x.junior@ufv.br (S.X.S.J.); 2 CJ do Brazil, Piracicaba 13432-700, São Paulo, Brazil; julianacristina_rezende@yahoo.com.br

**Keywords:** challenge, cytokines, L-tryptophan, immune response, performance, swine

## Abstract

This study aimed to evaluate the effects of the standardized ileal digestible tryptophan-to-lysine (SID Trp–Lys) ratio through the supplementation of different levels of L-tryptophan on pig performance and immune response following an LPS challenge. A total of 120 entire male pigs, with an average body weight of 16.5 ± 0.50 kg, were allocated in a randomized block design with four treatments, ten replicates per treatment, and three animals per experimental unit. The experimental treatments consisted of SID Trp–Lys ratios of 16%, 18%, 21%, and 24%, achieved through L-tryptophan supplementation. The evaluated performance parameters included the final body weight (BW), average daily gain (ADG), average daily feed intake (ADFI), and feed conversion ratio (FCR). Blood samples were collected on day 21 to determine serum serotonin levels, and on day 26, pigs were inoculated with LPS to induce an immune challenge, followed by blood sampling to assess cytokine responses. The results showed that pigs fed the 16% SID Trp–Lys ratio exhibited a lower FBW (*p* < 0.05). The SID Trp–Lys ratios influenced performance parameters, with quadratic responses (*p* < 0.05) observed for the FBW and FCR, where the highest FBW and lowest FCR were recorded at 22.05% and 21% SID Trp–Lys, respectively. A linear increase (*p* < 0.05) was observed for ADG, with a trend for a linear increase (*p* = 0.056) in ADFI. No effects (*p* > 0.10) of the SID Trp–Lys ratios were detected on serum serotonin levels. An increase in cytokine levels (GM-CSF, IFN-γ, IL-1α, IL-1β, IL-1ra, IL-4, IL-6, IL-8, IL-10, IL-12, IL-18, and TNF-α) was observed in pigs challenged with LPS (*p* < 0.10) compared to non-challenged animals. An interaction effect (*p* < 0.10) was detected for IL-2 and IL-18. SID Trp–Lys ratios between 21% and 24% optimize growth performance in pigs from 16 to 33 kg and modulate the immune response under LPS-induced challenge conditions.

## Simple Summary

Tryptophan (Trp) is an essential amino acid that pigs cannot synthesize naturally and must therefore be supplied through the diet. In addition to its role in protein synthesis, Trp contributes to serotonin production, which regulates appetite and other important physiological functions. Furthermore, Trp supports immune function and gut health. To maximize these benefits, an adequate proportion of Trp relative to lysine (Lys) is required. However, current recommendations for this proportion vary, indicating the need for further research. This study evaluated different proportions of Trp in relation to Lys and their effects on pigs challenged with *E. coli* lipopolysaccharide (LPS). The Trp proportion that optimized pig performance in this study was higher than the values reported in the literature, with 21% improving feed conversion and 24% enhancing weight gain and feed intake. Additionally, the 24% proportion favored a more efficient immune response under challenging conditions, such as LPS exposure. Therefore, the efficient use of Trp, combined with an adequate SID Trp–Lys ratio, can benefit the growth performance and health of pigs.

## 1. Introduction

Tryptophan (Trp) is an essential amino acid that must be supplied through the diet, as pigs lack the endogenous capacity to synthesize it. Like other essential amino acids, its limited availability compromises the efficient utilization of other amino acids for protein deposition. Beyond its role in protein synthesis, Trp is involved in the production of key metabolites, such as niacin (vitamin B3) and melatonin, a neurohormone derived from serotonin [1,2]. Serotonin, in turn, is a neurotransmitter with a significant role in regulating feed intake, sleep, body temperature, pain sensitivity, and aggressive behavior [3,4].

Additionally, Trp exerts beneficial effects on the immune system, including antioxidant capacity, maintenance of mucosal integrity, modulation of microbiota composition, and promotion of intestinal health [5]. These benefits are attributed to Trp catabolites, which activate the immune system through interactions with the aryl hydrocarbon receptor, expressed in various immune cells, including dendritic cells, macrophages, and lymphocytes [6,7]. Consequently, evaluating the immune response of pigs supplemented with Trp under challenging conditions can provide valuable insights into their ability to cope with infections and inflammatory processes. This information is crucial for determining appropriate dietary Trp levels in pig nutrition [8].

For proper Trp supplementation in starter pig diets, it is essential to establish the optimal standardized ileal digestible tryptophan-to-lysine (SID Trp–Lys) ratio. TBAS [9] recommend a ratio of 18%, whereas the NRC [10] suggests 16%, and TBAS [11] propose 19%. These varying recommendations highlight discrepancies in dietary guidelines. The increasing values proposed by TBAS [9] and TBAS [11] suggest a progressive upward adjustment of the SID Trp–Lys ratio over time, reinforcing the need for periodic updates.

Therefore, the hypothesis of this study is that higher levels of tryptophan than currently recommended may be necessary to optimize growth performance and enhance the resilience of growing pigs under immune challenge conditions. In this context, the objective was to evaluate the effects of the SID Trp–Lys ratio through L-tryptophan supplementation on pig performance and immune response following an LPS challenge.

## 2. Materials and Methods

The experiment was conducted at the Teaching, Research, and Extension Unit for Swine Production and Nutrition of the Department of Animal Science at the Federal University of Viçosa, located in Viçosa, Minas Gerais, Brazil.

### 2.1. Experimental Design, Animals, and Diets

The experiment was conducted with 120 non-castrated male pigs, commercial hybrids (Camborough × AGPIC 337, Agroceres PIC, Patos de Minas, MG, Brazil), with high genetic potential and an average body weight of 16.5 ± 0.50 kg. The animals were distributed in a randomized block design based on body weight, with four treatments, ten replicates per treatment, and three animals per pen, with the pen serving as the experimental unit.

During the experimental period, the animals were housed in suspended metal cages, equipped with semi-automatic feeders and nipple drinkers. Each pen housed three animals, with a space of 0.544 m^2^ per animal. Feed and water were provided ad libitum throughout the experiment. The room was equipped with electric heaters, and the temperature was adjusted to ensure optimal conditions.

The dietary treatments consisted of: T1 Basal diet deficient in tryptophan, corresponding to a SID Trp–Lys ratio of 16% without the addition of L-Tryptophan; T2 SID Trp–Lys ratio of 18%; T3 SID Trp–Lys ratio of 21%; and T4 SID Trp–Lys ratio of 24%. Tryptophan supplementation was carried out by adding L-Tryptophan-98%, supplied by CJ do Brasil (Piracicaba, São Paulo, Brazil).

The experimental diets were formulated based on corn and soybean meal and were properly supplemented with minerals and vitamins (Table 1). The amino acid levels, with the exception of Trp, were kept the same across all four treatments. The digestible lysine percentage in the diet was lower than the level recommended by the TBAS [11] for pigs in the early phase.

### 2.2. Performance Parameters

At the beginning and at the end of the experiment, all animals underwent weighing, and during the experimental period, the amount of feed provided and feed leftovers were quantified. Based on the collected data, the body weight (BW), average daily weight gain (ADG), average daily feed intake (ADFI), and feed conversion ratio (FCR) were calculated as responses to the different SID Trp–Lys dietary ratios.

### 2.3. Serotonin Analysis

On day 21 of the experimental period, blood samples were collected from 40 animals, with one animal randomly selected from each repetition, totaling 10 repetitions per treatment, to measure serotonin concentration. The collection was performed via the orbital sinus using tubes without anticoagulant, immediately placed on ice and then centrifuged at 1500× *g* at 4 °C (centrifuge R/5702, Copyright Eppendorf AG, Hamburg, Germany) to obtain serum samples. An enzyme-linked immunosorbent assay (ELISA) kit from Cloud-Clone Corp (Katy, TX, USA) was used to assess the serotonin levels in the obtained serum. The analyses were conducted by the Specialized Laboratory for Scientific Analyses (LEAC), located in Santana, São Paulo, Brazil.

### 2.4. Cytokine Analysis

On day 26 of the experiment, two animals were selected from six repetitions of each treatment for the immune challenge. The challenge consisted of the intramuscular (IM) administration of lipopolysaccharide (LPS) from Escherichia coli serotype O55:B5, provided by Sigma-Aldrich. For each repetition, one animal was chosen to receive the LPS dose and the other to receive sterile saline solution. The animal selected to receive LPS was administered a dose of 30 μg of LPS/kg of body weight, as per the methodology described by [12]. The animals assigned to the non-challenged group (control) received IM injections of sterile saline solution (0.9%) simultaneously with the animals challenged with LPS.

After the administration of the LPS solution or saline solution, a three-hour interval was observed, and blood collection was then performed. Blood was collected via the orbital sinus in tubes without anticoagulant. The tubes were immediately placed in a container with ice and later centrifuged to obtain serum. The samples were centrifuged at 1900× *g* at 4 °C (centrifuge R/5702, Copyright Eppendorf AG, Hamburg, Germany). Subsequently, the serum samples were stored in Eppendorf tubes and kept at −20 °C to preserve sample integrity until analysis. A MILLIPLEX^®^MAP kit (Porcine Cytokine/Chemokine Magnetic Bead Panel Cat#PCYTMAG-23K, Darmstadt, Germany) was used to assess the inflammatory response of the Granulocyte and Macrophage Colony-Stimulating Factor (GM-CSF), Interferon-Gamma (IFNγ), Interleukin-1 Alpha (IL-1α), Interleukin-1 Beta (IL-1β), Interleukin-1 Receptor Antagonist (IL-1ra), Interleukin-2 (IL-2), Interleukin-4 (IL-4), Interleukin-6 (IL-6), Interleukin-8 (IL-8), Interleukin-10 (IL-10), Interleukin-12 (IL-12), Interleukin-18 (IL-18), and Tumor Necrosis Factor (TNFα). The analyses were performed at the Laboratory of Specialized Scientific Analyses (LEAC), located in Santana, São Paulo, Brazil.

### 2.5. Statistical Analysis

All statistical analyses were performed using R software, version 4.3.1 (RStudio, 2023). The performance data were subjected to analysis of variance (ANOVA), considering significant differences at (*p* < 0.05), with the pen as the experimental unit. Orthogonal polynomial contrasts were applied to identify the linear and quadratic effects of the Trp DIE–Lys DIE ratios. Serotonin levels were also analyzed by ANOVA (*p* < 0.05), considering the fixed effects of diet and block, with the animal from the repetition as the experimental unit. For cytokines, significance was considered when (*p* < 0.10), including the effects of diet, LPS challenge, and block in the model.

## 3. Results

### 3.1. Performance

The performance results are presented in Table 2. The analysis of the animals’ initial body weight showed no statistically significant differences, indicating sample homogeneity and confirming no impact on the obtained results.

The effects of the SID Trp–Lys ratios on the final body weight (FBW), ADG, and FCR were observed (*p* < 0.05). The increase in the SID Trp–Lys ratio in the pigs’ diet resulted in a quadratic response in the animals’ FBW. Based on the equation values, the maximum FBW for the SID Trp–Lys ratio was calculated, reaching FBW = 32.709 kg when X = 22.05, where X represents the SID Trp–Lys ratio, according to the quadratic polynomial regression model.

The SID Trp–Lys ratios showed a progressive increase in ADG and ADFI (*p* < 0.05) following a linear trend, reaching the highest tested ratio of 24% (ADG = 0.777; ADFI = 1.184).

There was a significant difference (*p* = 0.032) for the values obtained for FCR, where the lowest SID Trp–Lys ratio of 16% promoted the highest value (FCR = 1.585), while the 21% ratio led to the lowest value (FCR = 1.509). The results were significant for both linear and quadratic equations. The minimum value calculated for the FCR was estimated at FCR = 1.505 when X = 21.15, considering the SID Trp–Lys ratio according to the quadratic polynomial regression model.

### 3.2. Serotonin

The effects of the SID Trp–Lys ratio on serotonin levels are presented in Table 3. No differences were observed (*p* > 0.05) between the levels, indicating that the SID Trp–Lys dietary ratios did not influence serotonin levels.

### 3.3. Cytokines

Table 4 presents the results of blood cytokine concentrations in pigs, with or without *E. coli* LPS challenge, after consuming diets with different SID Trp–Lys ratios. All results were significant (*p* < 0.10) for the response to the challenge. However, there was no significant independent effect (*p* < 0.10) for the SID Trp–Lys levels on the response of the evaluated cytokines. Interaction effects were observed between the SID Trp–Lys ratios and the immune challenge on the concentrations of IL-2 (*p* = 0.050) and IL-18 (*p* = 0.064) presented in Table 5.

The breakdown of IL-2 showed that the SID Trp–Lys ratio significantly influenced the results of the control group (*p* = 0.009), with the highest IL-2 response observed at the 21% SID Trp–Lys ratio. In the group of animals challenged with LPS, there was no significant difference between the SID Trp–Lys ratios (*p* = 0.789). However, there was a significant difference (*p* = 0.033) between the control and LPS groups at the 24% SID Trp–Lys ratio, where the IL-2 response was higher in the LPS-challenged group (0.182) compared to the control group (0.065).

The SID Trp–Lys ratio significantly influenced the IL-18 results in both the control and LPS-challenged groups. In the control group, there was no significant difference in the SID Trp–Lys ratio on the IL-18 response (*p* > 0.10). However, in the LPS-challenged group, the 24% SID Trp–Lys ratio resulted in the highest IL-18 response (1.417), with significance (*p* = 0.076). A significant difference was observed for the 16% (*p* = 0.024), 18% (*p* = 0.003), and 24% (*p* < 0.001) SID Trp–Lys ratios, which showed an increase in IL-18 concentration when challenged with LPS.

## 4. Discussion

Ensuring precise feeding that meets the nutritional requirements of pigs in the early phase is crucial from both an economic and environmental perspective in animal production, including the determination of the ideal tryptophan-to-lysine ratio.

The study demonstrated that the standardized ileal digestible tryptophan-to-lysine ratio influenced the final BW of the animals, indicating a quadratic response, with the maximum calculated value for the final BW being 32.709 when X = 22.05, according to the quadratic polynomial regression model. This is the ideal SID Trp–Lys ratio for pigs in the early phase to achieve the highest final BW.

A study evaluated different SID Trp–Lys ratios for pigs in the early phase and found a higher final BW with the maximum supplementation of 23% [13]. However, the Linear-Plateau Response Model (LRP) showed that the maximum final BW was achieved when the ratio was 22%, consistent with the findings of this study. The maximum BW of animals associated with higher levels of Trp supplementation may indicate efficient nutrient use and a limiting effect of Trp on the development of animals with low SID Trp–Lys ratios. Other authors also reported that lower Trp levels were correlated with a lower final BW of the animals [14,15,16].

According to [17], a SID Trp–Lys ratio of 23% and 24% in the diet of pigs in the early phase promoted higher ADG, which is consistent with the findings of this study, where at the highest level of 24%, the animals exhibited the highest ADG, with a linear effect. In another study, a meta-analysis conducted by [18] demonstrated that increasing the SID Trp–Lys from 17% to 22% increased ADG by 8%, a significant value in pig production. These studies confirm that Trp has an influence on the animals’ weight gain, which may be related to consumption.

The increase in intake also showed a linear relationship with the levels of Trp in the diet. In the present study, a 5% increase in intake was observed when the Trp levels were raised from 16% to 24%, in line with previous findings by [15,19,20], who also recorded a linear effect of Trp levels on feed intake. This effect can be explained by the influence of the ideal amounts of Trp on increased serotonin production, stimulating feed intake [2]. Furthermore, Trp deficiency can reduce feed intake in piglets due to the increase in neutral amino acids such as valine, leucine, isoleucine, and phenylalanine, which may interfere with the availability of Trp for serotonin production, thus reducing feed intake [14].

However, in this study, no effect of the SID Trp–Lys ratio on serotonin concentrations was observed. Ref. [21] investigated the effects of the SID Trp–Lys ratio on serotonin levels in plasma and the hypothalamus. The results indicated that plasma serotonin concentrations could not be explained by Trp levels, while the concentration in the hypothalamus increased linearly with the increase in tryptophan levels. In a study conducted by [22], the authors demonstrated that serotonin responses were associated with Trp levels when evaluated in the hypothalamus. These results suggest that the hypothalamus may provide a more accurate indication of serotonin concentration compared to blood parameters.

Additionally, the lack of effect observed may be attributed to the timing of data collection. In the present study, serotonin levels were evaluated at 21 days of the experiment. It is plausible to consider that, by this time, the animals may have already adapted to the Trp levels present in the diet, which could have mitigated any measurable response to the increase or decrease in serotonin levels in response to the SID Trp–Lys ratio.

The increase in intake is particularly relevant, especially since animals in the early stages typically reduce intake in the first weeks after weaning, impairing performance. The stimulation of intake provided by Trp supplementation may play a key role in improving the performance of pigs during the early growth phase, as well as in subsequent phases. Therefore, future research is needed to investigate the effects of the SID Trp–Lys ratio at different time points during the experiment, allowing for a more comprehensive understanding of the effects of Trp on serotonin regulation and, consequently, on the feeding behavior of pigs.

The literature provides different recommendations for the SID Trp–Lys ratio. For example, the NRC [10] suggests a ratio of 16%, while TBAS [11] recommend 19% for pigs in the early phases, which are the main references used in formulating diets. In previous studies [16,23,24,25], ideal values of 15%, 16%, 17%, and 19% were found, respectively. However, these values are below the ideal ratio identified in this study, which was 21% to achieve the minimum CA value, a crucial parameter for determining animal performance. More recent studies, such as the one by [17], support the results of this study, also finding better CA with 21%. Our findings suggest an increase in the Trp requirement in the diet and, consequently, the need to adjust the SID Trp–Lys ratio. This result is possibly related to the advancement of the genetic potential of the animals and the environmental conditions in which they are placed. Immunological challenge conditions also increase the requirement for amino acids, including Trp [26,27]. This happens because, in the presence of pathogens and unfavorable sanitary conditions, the metabolism of Trp is modified, redirecting it from growth functions to immune support functions.

Our study aimed to evaluate the acute inflammatory response in pigs in the early phase, seeking to understand how these animals would respond to a challenge after being previously supplemented with diets containing different levels of Trp. The hypothesis was that animals supplemented with higher levels of Trp would show a better immune response to the applied challenge, due to a more robust immunity established by Trp supplementation.

The elevation of cytokines after the LPS challenge in this study confirmed the effectiveness of the proposed model in activating the signaling pathways responsible for the secretion of cytokines in response to LPS-induced inflammation [28,29]. As expected, there was an increase in pro-inflammatory cytokines, which play a key role in the immune response, being rapidly released in the presence of LPS, found in the cell wall of Gram-negative bacteria [30].

Studies conducted by [31,32] investigated the effect of Trp on the immune response of pigs challenged with LPS. They reported a significant reduction in the cytokines IL-1β, IL-6, IL-8, and TNF-α, evaluated in the liver and colon of the pigs. These findings suggest that the inclusion of Trp in the diet may have mitigated the inflammatory response triggered by LPS. However, in our study, we observed different responses, where higher levels of tryptophan were generally associated with a greater expression of cytokines. While a lower inflammatory response is typically considered beneficial, due to the reduction in feed intake and the reallocation of nutrients intended for animal growth [33,34], the absence of an inflammatory response could also indicate that the animal’s immune system is not properly prepared to handle the challenge, which may negatively impact its future performance. Particularly in acute challenge situations, as proposed in our study, it is crucial that cytokines are activated rapidly and efficiently to regulate inflammation and ensure an adequate immune response.

Our study demonstrated significant interactions for the interleukins IL-2 and IL-18 in pigs subjected to an LPS challenge after consuming diets with a different SID Trp–Lys ratio. These interactions are crucial for understanding how different dietary amino acid proportions can modulate the immune response. In the control group, the animals supplemented with a 21% SID Trp–Lys ratio exhibited higher IL-2 production, indicating an enhanced immune response under normal conditions. This result is consistent with the performance outcomes of the animals in the performance study, suggesting a possible correlation between increased immune response and better feed efficiency, indicating that the animals were in a better immunological condition. During the response to the challenge, the highest IL-2 expression was observed in the group challenged with LPS and supplemented with a 24% SID Trp–Lys ratio, suggesting that the 24% ratio is more effective under challenging conditions. IL-2 plays a crucial role in the maturation of B and T lymphocytes and the regulation of leukocyte functions, which are essential for immunity.

The SID Trp–Lys ratio and the challenge influenced the IL-18 results. IL-18 is crucial for initiating and regulating inflammatory responses, particularly under immune stress, promoting the production of pro-inflammatory cytokines and activating immune cells. In the control group, there was no significant difference in Trp levels regarding the IL-18 response, suggesting that, in the absence of immune stress, variations in the Trp and lysine ratio in the diet do not affect IL-18 production. However, in the group challenged with LPS, the 24% SID Trp–Lys ratio resulted in the highest IL-18 response. This is consistent with the results from [32], who observed a significant linear increase in IL-18 with the addition of 0.2% and 0.4% Trp in the diet of LPS-challenged piglets.

The significant interaction of interleukins IL-2 and IL-18 suggests that higher levels of the SID Trp–Lys ratio modulate the immune response in pigs, especially under the LPS challenge. These findings have practical implications for diet formulation that enhances the immune health of pigs, helping them better respond to common challenges in production and increasing resilience in stressful situations.

In addition, the lack of significant results in this study may be attributed to some limitations of the experiment. Only the acute cytokine response was analyzed, without considering the recovery of the animals after the inflammatory challenge. It is possible that the animals responded differently during the recovery phase, which could influence the results, making it relevant for the choice of the SID Trp–Lys ratio.

## 5. Conclusions

Considering that the SID Trp–Lys ratios that optimize piglet performance are higher than those suggested in the literature, a SID Trp–Lys ratio of 21% for FCR and 24% to achieve the best ADG and ADFI responses is recommended. Furthermore, the 24% SID Trp–Lys ratio may provide a better immune response when animals are subjected to challenges.

Additionally, it is evident that further studies are needed for a more in-depth understanding of the immune response in animals and the effects of the SID Trp–Lys ratio on serotonin levels. These investigations could provide valuable insights for formulating more effective diets and promoting the well-being and health of pigs.

## Figures and Tables

**Table 1:** Experimental diets.

	SID Trp–Lys Ratio			
Ingredients	16%	18%	21%	24%
Corn 7.88%	65.54	65.54	65.54	65.54
Soybean Meal 45%	28.28	28.28	28.28	28.28
Soybean Oil	1.05	1.05	1.05	1.05
Dicalcium Phosphate	1.92	1.92	1.92	1.92
Calcitic Limestone	0.87	0.87	0.87	0.87
Salt	0.48	0.48	0.48	0.48
Vitamin Premix ^1^	0.30	0.30	0.30	0.30
Mineral Premix ^2^	0.25	0.25	0.25	0.25
L-Lysine HCL	0.49	0.49	0.49	0.49
L-Threonine	0.25	0.25	0.25	0.25
L-Methionine	0.21	0.21	0.21	0.21
L-Valine	0.11	0.11	0.11	0.11
Antioxidant Banox	0.01	0.01	0.01	0.01
Kaolin	0.20	0.17	0.13	0.09
L-Tryptophan ^3^	0.00	0.02	0.06	0.10
Total	100.00	100.00	100.00	100.00
Expected Nutritional Values				
Metabolizable Energy (kcal/kg)	3239.20	3239.20	3239.20	3239.20
Net Energy (kcal/kg)	2470.00	2470.00	2470.00	2470.00
Ether Extract %	4.09	4.09	4.09	4.09
Crude Fiber %	2.50	2.50	2.50	2.50
Neutral Detergent Fiber %	12.89	12.89	12.89	12.89
Acid Detergent Fiber %	4.26	4.26	4.26	4.26
Calcium %	0.91	0.91	0.91	0.91
Phosphorus %	0.67	0.67	0.67	0.67
Available Phosphorus %	0.45	0.45	0.45	0.45
Potassium %	0.72	0.72	0.72	0.72
Sodium %	0.20	0.20	0.20	0.20
Chlorine %	0.36	0.36	0.36	0.36
Crude Protein %	18.82	18.82	18.82	18.82
Crude Protein Dig. %	16.21	16.21	16.21	16.21
Lysine Dig. %	1.21	1.21	1.21	1.21
Methionine Dig. %	0.46	0.46	0.46	0.46
Methionine + Cystine Dig. %	0.73	0.73	0.73	0.73
Threonine Dig. %	0.83	0.83	0.83	0.83
Tryptophan Dig. %	0.19	0.21	0.25	0.29
Arginine Dig. %	1.10	1.10	1.10	1.10
Valine Dig. %	0.88	0.88	0.88	0.88
Isoleucine Dig. %	0.68	0.68	0.68	0.68
Leucine Dig. %	1.44	1.44	1.44	1.44
Histidine Dig. %	0.44	0.44	0.44	0.44
Phenylalanine Dig. %	0.81	0.81	0.81	0.81

^1^ Provided the following quantities per kilogram of complete diet: Se, sodium selenite and selenium yeast, 75.00 mg/kg; vitamin A, retinyl acetate, 2000.00 IU/kg; vitamin D3, cholecalciferol, 375,000.00 IU/kg; vitamin E, DL-alpha tocopherol, 6250.00 IU/kg; vitamin K3, bisulfate of menadione nicotinamide, 750.00 mg/kg; thiamine, thiamine mononitrate, 500.00 mg/kg; riboflavin, 1500.00 mg/kg; pyridoxine, pyridoxine hydrochloride, 500.00 mg/kg; vitamin B12, 7500.00 mcg/kg; folic acid, 250.00 mg/kg; pantothenic acid, D-calcium pantothenate, 5000.00 mg/kg; niacin, 8750.00 mg/kg; biotin, 37.50 mg/kg. ^2^ Provided the following quantities per kilogram of complete diet: Fe, 15.00 g/kg iron sulfate; Cu, 40.00 g/kg copper sulfate; Mn, 13.00 g/kg manganese monoxide; Zn, 25.00 g/kg zinc sulfate; I, 350.00 mg/kg calcium iodate. ^3^ L-tryptophan was provided by CJ do Brasil (Piracicaba, São Paulo, Brazil).

**Table 2:** Performance of growing pigs receiving diets with different SID Trp–Lys ratios obtained from L-Tryptophan supplementation.

Parameters	SID Trp–Lys Ratios						Polynomial Contrasts ^1^	
							LPR	QPR
	16%	18%	21%	24%	ANOVA *p*-Values	SEM	*p*-Value	
Initial BW, kg	16.310	16.690	16.630	16.560	0.241	0.085	0.276	0.154
Final BW, kg	30.731	32.685	32.203	32.709	<0.001	0.172	<0.001	0.019
ADG, kg/day	0.691	0.769	0.742	0.770	<0.001	0.007	<0.001	0.142
ADFI, kg/day	1.121	1.179	1.147	1.184	0.031	0.009	0.056	0.639
FCR	1.585	1.533	1.509	1.529	0.032	0.010	0.044	0.029

The tested model is significant *p* < 0.10. Abbreviations: Standard Error of the Mean (SEM); linear polynomial regression model (LPR), quadratic polynomial regression model (QPR); body weight (BW); average daily gain (ADG); average daily feed intake (ADFI); feed conversion ratio (FCR). ^1^ Polynomial contrasts: final BW = 28.4219 + 0.1850 Trp; final BW = 10.5808 + 2.0093 Trp − 0.0456 Trp^2^; ADWG = 0.5966 + 0.0075 Trp; ADFI = 1.0480 + 0.0055 Trp; FCR = 1.6629 − 0.0062 Trp and FCR = 2.8157 − 0.1238 + 0.0029 Trp^2^.

**Table 3:** Serum serotonin concentration in growing pigs receiving diets with different SID Trp–Lys ratios obtained from L-tryptophan supplementation.

	SID Trp–Lys Ratios				ANOVA		*p*-Value	
Parameters	16%	18%	21%	24%	*p*-Value	SEM	LPR	QPR
Serotonin ng/mL	237.73	300.67	208.49	268.41	0.193	15.912	0.903	0.778

The tested model is significant *p* < 0.5. Abbreviations: Standard Error of the Mean (SEM); linear polynomial regression model (LPR); quadratic polynomial regression model (QPR); data collected from 40 animals.

**Table 4:** Concentration of blood cytokines in pigs challenged or not with *E. coli* LPS after consuming diets with different SID Trp–Lys ratios obtained from L-tryptophan supplementation during growth.

	SID Trp–Lys Ratios									*p*-Value		
	LPS ^1^				CON							
Parameters ng/mL	16%	18%	21%	24%	16%	18%	21%	24%	SEM	Level	Challenge	L*C ^2^
GM-CSF	0.045	0.042	0.045	0.051	0.027	0.021	0.023	0.025	0.003	0.879	<0.001	0.962
IFN γ	0.209	0.383	0.264	0.215	0.153	0.100	0.185	0.047	0.024	0.207	<0.001	0.157
IL-1α	0.043	0.047	0.043	0.059	0.008	0.005	0.015	0.006	0.005	0.597	<0.001	0.137
IL-1β	1.041	0.788	1.144	1.347	0.049	0.034	0.049	0.039	0.098	0.425	<0.001	0.443
IL-1ra	77.931	70.033	68.587	70.020	0.325	0.241	0.313	0.255	5.410	0.349	<0.001	0.358
IL-2	0.160	0.197	0.153	0.182	0.096	0.119	0.225	0.065	0.014	0.225	0.065	0.050
IL-4	0.101	0.139	0.137	0.199	0.079	0.064	0.156	0.069	0.013	0.323	0.037	0.158
IL-6	9.323	8.369	10.105	11.410	0.129	0.088	0.186	0.109	0.841	0.654	<0.001	0.668
IL-8	2.793	3.973	3.442	4.029	0.154	0.087	0.131	0.232	0.320	0.644	<0.001	0.674
IL-10	1.643	2.113	2.058	2.287	0.121	0.099	0.421	0.079	0.145	0.310	<0.001	0.107
IL-12	3.710	3.340	4.630	3.700	2.150	2.250	2.230	2.420	0.169	0.204	<0.001	0.169
IL-18	0.859	1.027	0.913	1.417	0.312	0.346	0.638	0.311	0.075	0.337	<0.001	0.064
TNFα	0.546	0.587	1.049	0.935	0.023	0.025	0.032	0.032	0.077	0.171	<0.001	0.176

The tested model is significant *p* < 0.10. ^1^ The *E. coli* lipopolysaccharide (LPS) was used to induce an inflammatory response in the group challenged with LPS. The challenge consisted of a dose of 30 μg/kg of LPS. The control group received saline solution (0.9%). The LPS solution and saline solution were administered on day 26 of the experiment, after the performance data collection. ^2^ L*C interaction effect of level and challenge. Standard Error of the Mean (SEM); Control (CON); Granulocyte-Macrophage Colony-Stimulating Factor (GM-CSF); Interferon-Gamma (IFN γ); Interleukin-1 Alpha (IL-1α); Interleukin-1 Beta (IL-1β); Interleukin-1 Receptor Antagonist (IL-1ra); Interleukin-2 (IL-2); Interleukin-4 (IL-4); Interleukin-6 (IL-6); Interleukin-8 (IL-8); Interleukin-10 (IL-10); Interleukin-12 (IL-12); Interleukin-18 (IL-18); and Tumor Necrosis Factor (TNFα).

**Table 5:** Unfolding of the interaction between the SID Trp–Lys ratios and animals challenged or not with E. coli LPS on the concentration of IL-2 and IL-18, 3 h after the challenge.

Challenge	SID Trp–Lys Ratios				*p*-Value
	16%	18%	21%	24%	
	IL-2 ng/mL Response				
CON	0.099 b	0.119 ab	0.225 a	0.065 b	0.009
LPS	0.160	0.197	0.153	0.182	0.789
*p*-value	0.201	0.109	0.138	0.033	
	IL-18 ng/mL Response				
CON	0.312	0.346	0.638	0.312	0.427
LPS	0.859 b	1.027 ab	0.913 ab	1.417 a	0.076
*p*-value	0.024	0.003	0.203	<0.001	

Means in the row followed by distinct letters differ by the Tukey test (*p* < 0.10); for means in the columns, observe the *p*-value for the contrasts presented. The *E. coli* lipopolysaccharide (LPS) and control (CON).

## Data Availability

The data presented in this study are available on request from the corresponding author.
